# Assessment of new-onset heart failure prediction in a diabetic population using left ventricular global strain: a prospective cohort study based on UK Biobank

**DOI:** 10.3389/fendo.2024.1365169

**Published:** 2024-04-02

**Authors:** Siwei Chen, Cong Chen, Longxuan Zheng, Wenke Cheng, Xiancong Bu, Zhou Liu

**Affiliations:** ^1^ Department of Cardiovascular Medicine, The Third Hospital of Nanchang, Jiangxi, China; ^2^ Department of Cardiology, Zaozhuang Municipal Hospital, Zaozhuang, China; ^3^ Department of Cardiology, The Fifth People’s Hospital of Huai’an, The Affiliated Huai’an Hospital of Yangzhou University, Huai’an, China; ^4^ Medical Faculty, University of Leipzig, Leipzig, Germany; ^5^ Department of Neurology, Zaozhuang Municipal Hospital, Zaozhuang, China; ^6^ Department of Geriatric Medicine/Cardiology, The Fifth People’s Hospital of Huai’an, The Affiliated Huai’an Hospital of Yangzhou University, Huai’an, China

**Keywords:** global radial strain, global circumferential strain, global longitudinal strain, heart failure, UK biobank

## Abstract

**Background:**

Impaired glucose utilization influences myocardial contractile function. However, the prognostic importance of left ventricular global radial strain (LV-GRS), left ventricular global circumferential strain (LV-GCS), and left ventricular global longitudinal strain (LV-GLS) in predicting new-onset heart failure (HF) in a population with diabetes is unclear.

**Methods:**

The study design is prospective cohort from the UK Biobank. Totally 37,899 participants had a complete data of cardiac magnetic resonance (CMR), of which 940 patients with diabetes were included, and all the participants completed follow-up. LV-GRS, LV-GCS, and LV-GLS were measured by completely automated CMR with tissue tagging. Cox proportional hazards regression analysis and C-index was performed to evaluate the association between the strain parameters and the new-onset HF in patients suffering from diabetes.

**Results:**

The average age of the 940 participants was 57.67 ± 6.97 years, with males comprising 66.4% of the overall population. With an average follow-up period of 166.82 ± 15.26 months, 35 (3.72%) patients reached the endpoint (emergence of new-onset HF). Significant associations were found for the three strain parameters and the new-onset HF (LV-GRS—hazard ratio [HR]: 0.946, 95% CI: 0.916-0.976; LV-GCS—HR: 1.162, 95% CI: 1.086-1.244; LV-GCS—HR: 1.181, 95% CI: 1.082-1.289). LV-GRS, LV-GCS, and LV-GLS were closely related to the related indicators to HF, and showed a high relationship to new-onset HF in individuals with diabetes at 5 and 10 years: LV-GRS: 0.75 (95% CI, 0.41-0.94) and 0.76 (95% CI, 0.44-0.98), respectively; LV-GCS: 0.80 (95% CI, 0.50-0.96) and 0.75 (95% CI, 0.41-0.98), respectively; LV-GLS: 0.72 (95% CI, 0.40-0.93) and 0.76 (95% CI, 0.48-0.97), respectively. In addition, age, sex, body mass index (BMI), and presence of hypertension or coronary artery disease (CAD) made no impacts on the association between the global strain parameters and the incidence of HF.

**Conclusion:**

LV-GRS, LV-GCS, and LV-GLS is significantly related to new-onset HF in patients with diabetes at 5 and 10 years.

## Graphical Abstract

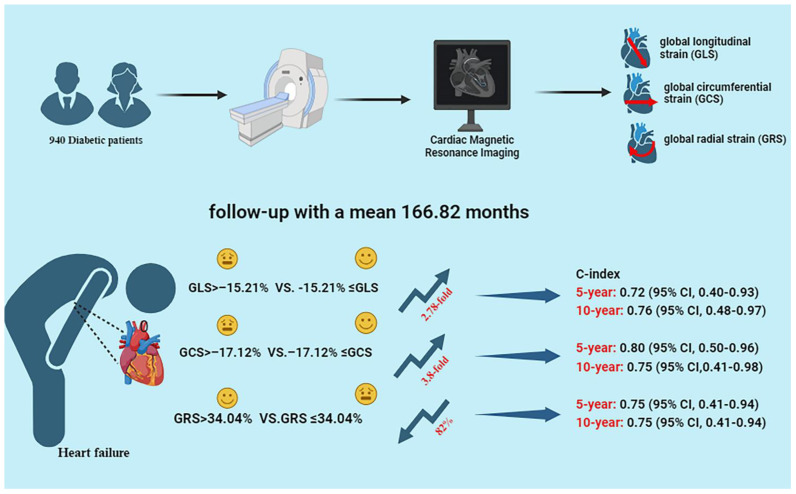

940 diabetic patients completed cardiac MRI including global longitudinal strain (GLS), global circumferential strain (GCS), and global radial strain (GRS). After a mean follow-up of 166.82 months, 35 patients developed heart failure (HF). The results showed that GRS, GCS and GLS were significantly associated with new-onset HF in diabetic patients, with a 3.8-fold and 2.78-fold increase in the risk of HF in the high GCS and GLS groups, respectively, and an 82% reduction in the risk of HF in the high GRS group. Global left ventricular strain was highly predictive of new-onset HF at 5 and 10 years, with a C-index of more than 75%.

## Introduction

Diabetes mellitus is a serious health problem characterized by insufficient insulin secretion or a weakened body response to insulin, causing dysregulation of carbohydrate metabolism, which in turn leads to a sustained increase in blood glucose levels (1). In patients with diabetes, insulin-stimulated uptake and utilization of glucose are impaired and energy metabolism in cardiomyocytes is altered due to the defects in insulin-mediated actions or insulin resistance (2). Increasing epidemiological evidence suggests that diabetes mellitus is independently associated with the risk of developing HF, with the risk being four times higher in patients with diabetes than in the normal population (3). In a study carried out by Nichols et al. among 8231 participants with diabetes (none of whom had HF at baseline) and 8845 participants without diabetes, the incidence of HF was 12.4 per 1000 person-years in participants without diabetes, while the incidence was 30.9 per 1000 person-years in participants with diabetes (4). Various mechanisms result in impaired systolic and diastolic function of the heart in patients with diabetes, including abnormal processing of glucose and free fatty acids (FFAs) by the heart, and the impact of metabolic disorders on the cardiovascular system, all contributing to the increased risk of HF in patients with diabetes (3). Epidemiological and clinical data collected over the past 20 years indicate that patients with diabetes develop HF irrespective of the existence of coronary artery disease (CAD) or its related risk factors (5). Compared with normal healthy participants, differences in mitral valve flow patterns indicating diastolic dysfunction were observed in patients with diabetes, while no significant differences in global systolic function as determined by ejection fraction (EF) were found between the two groups (6, 7), suggesting that assessing the cardiac systolic function based on left ventricular ejection fraction (LVEF) may miss a subset of patients who could be benefited from early intervention to prevent adverse outcomes of the heart in the future. Therefore, strain imaging may become a more sensitive indicator to assess myocardial deformation.

Myocardial strain is a key indicator of myocardial deformation and can be obtained by cardiac magnetic resonance (CMR) imaging. CMR imaging refers to a non-invasive diagnostic technique using a magnetic field and radiofrequency waves to produce detailed images of the heart and surrounding structures. Myocardial strain is consisted of three parameters including left ventricular global longitudinal strain (LV-GLS), left ventricular global circumferential strain (LV-GCS) and left ventricular global radial strain (LV-GRS). Strain imaging allows for rapid and accurate analysis of global and regional left ventricular (LV) systolic and diastolic mechanics in the longitudinal, radial, and circumferential strain in multiple segments and rotational parameters (8). Compared with speckle tracking echocardiography, the strain measured by CMR is gradually gaining popularity due to its high spatial and high tissue resolution (9–13). In the evaluation of HF, myocardial regional and global systolic and diastolic functions indicated by LV strain are key measurements (14–16). Particularly, assessing myocardial strain using CMR tagging is the noninvasive gold standard for quantitative assessment of segmental wall motion abnormalities of the left ventricle (17). However, CMR is usually recommended for those patients experiencing high-grade or complex cardiac disease, especially after completion of an initial first-line diagnosis, including transthoracic echocardiography. In addition, considering the relatively high cost of CMR, its popularity in the clinical setting is limited and its use varies significantly from region-to-region (18). These factors contribute to the relatively rare use of CMR in the general population, which can therefore limit our full understanding of its effectiveness in this population.

It is demonstrated that even if the left ventricular ejection fraction (LVEF) remains normal, the early stages of ventricular disease may show defects in LV systolic function (8, 9). Moreover, studies have indicated that impaired systolic function is associated with diabetes mellitus (19–21). However, till the present, no studies have evaluated the relationship between strain imaging and the risk of developing HF in the future in patients with diabetes. We hypothesized that cardiac strain might be capable of detecting subtle changes in ventricular function earlier in the diabetic population, thereby compensating for the limitations of LVEF in diagnosing early ventricular dysfunction. Measuring systolic function by CMR in diabetic patients may identify individuals at high risk of HF, which may be beneficial for patients requiring enhanced clinical monitoring and changes in the current therapies. The UK, with one of the highest rates of CMR adoption globally, provided the setting for this study (18). Therefore, based on the UK Biobank, this study aimed to investigate the relationship between myocardial strain and new-onset HF in diabetic patients.

## Methods

### Study design and population

The UK Biobank, established between 2006 and 2010, is a large prospective cohort and represents a comprehensive biomedical research database, consisting of 502,000 adults aged between 40 and 69 years from England, Scotland, and Wales (22). The UK Biobank is an extensive collection of genetic, physiological, and lifestyle data. The health outcomes of participants are systematically monitored based on data from electronic health records from Hospital Episode Statistics, cancer registry data, and death registers. A detailed description of the study design and methods can be found in a previously published study (23).

In this study, totally 37,899 participants had a complete CMR data, of which 1,017 patients with diabetes were identified by self-report or medical records. Then, participants with pre-existing HF (n=5) and cancer (n=72) at baseline were excluded. A total of 940 individuals with diabetes were included in the current analyses. These participants were nonpregnant and had complete follow-up data. All participants had a follow-up period of more than two years, which contributed to reducing the possibility of reverse causation. Ethical approval for the UK Biobank was obtained from the North West Multicenter Research Ethics Committee, Manchester, UK (REC reference: 11/NW/0382). All participants provided written informed consent.

### Assessment of cardiovascular magnetic resonance

CMRI was employed to assess the cardiovascular structure and function of the participants. The CMR images were captured using 1.5T scanners (MAGNETOM Aera, Syngo Platform VD13A, Siemens Healthcare, Erlangen, Germany). CMRI is one of the imaging modalities used by the UK Biobank to collect data on various health-related factors from participants (24). CMRI scan was performed by a radiographer who had been provided with suitable training and had been granted the relevant module permissions.

CMR were assessed for image quality and artifacts by a senior radiographer. In addition, the acquisition protocol is also published elsewhere (25). The CMRI acquisitions include piloting and sagittal, transverse, and coronal partial coverage of the chest and abdomen. Meanwhile, three long-axis cines (the apical two-chamber, three-chamber, and the apical four-chamber) and complete short-axis images were collected to assess left ventricle function based on the following parameters: left ventricular ejection fraction (LVEF), left ventricular mass index (LVMS), left ventricular end-systolic volume (LVESV), left ventricular end-diastolic volume (LVEDV), left ventricular mean myocardial wall thickness, and measures of cardiac LV strain, including LV-GRS, LV-GCS, and LV-GLS by CMR tissue tagging. The CMRI indices were obtained through a completely automated quality-controlled image analysis pipeline that had been previously developed and validated in a significant subset of the UK Biobank (26, 27).

### Assessment of diabetes mellitus

Diabetes was diagnosed based on participants’ self-reported diagnoses, medication use, and hospital records. Participants were asked, “Has a doctor ever told you that you have diabetes?” and “ What was your age when the diabetes was first diagnosed?” A positive response to these questions suggested a self-diagnosis of diabetes. Medical records, including both hospital and primary care history, and use of antihyperglycemic medications were employed to further confirm the diagnosis of diabetes. Participants who had a documented medical diagnosis of diabetes before entry into the cohort were also classified as having diabetes.

### Assessment of heart failure

The UK Biobank combines multiple data sources, including death registries, primary care records, hospitalization admission records, and self-reports, to create health condition-specific diagnostic algorithms, aiming to accurately identify specific health conditions. Individuals who were free of HF at baseline but who subsequently had any hospital records indicative of HF based on the International Classification of Diseases codes, Tenth Revision (ICD-10) were considered to have had the outcome of interest. To identify the date and diagnosis of hospital admissions, Hospital Episode Statistics in England and Wales and Scottish Morbidity Records in Scotland were linked to the UK Biobank. Incident HF was ascertained as a hospital admission with code of I50. Date of the first record labeled as code I50 was identified as the date of the initial diagnosis of HF for that participant. Data were continuously updated for all the participants. The date of the last reported case of HF (05 December 2022) marked the end of the follow-up period for this study.

### Assessment of other variables

The demographic and medical information of participants was collected. Self-reported information included age, gender, race, education level, smoking and drinking status, and information on diabetes medication uses and age at the onset of diabetes. BMI was calculated from the height and weight measurements taken during the participants’ initial visit to the assessment center. Lipid levels were evaluated using an enzymatic assay on the Beckman Coulter AU5800 instrument (Beckman Coulter (UK), Ltd). HbA1c levels were measured using high-performance liquid chromatography on a Bio-Rad Variant II Turbo analyzer (Bio-Rad Laboratories, Inc.). Using the manufacturer’s reagents and calibrators, serum creatinine was measured by an enzymatic method on a Beckman Coulter AU5400 clinical chemistry analyzer. The estimated glomerular filtration rate (eGFR) was calculated using the Chronic Kidney Disease Epidemiology Collaboration (CKD-EPI) equation (28). Blood pressure was recorded using an automated sphygmomanometer at the assessment center; in cases where the automated device was not suitable, a manual sphygmomanometer was applied. The diagnosis of CAD and hypertension was based on patients’ self-reports and medical records. Participants who reported having experienced a myocardial infarction or angina were considered to have CAD. Participants who were taking antihypertensive medication were classified as hypertensive. Moreover, participants who did not self-report and had a clear medical diagnosis documented before entry into the cohort were identified as having CAD and hypertension.

### Statistical analyses

R 4.2.2 software was used to conduct the statistical analysis. The statistical analysis was performed using R 4.2.2 software. Multiple imputation (10 data sets) was performed using the ‘mice’ package to address missing covariates. The proportion of missing data would make an impact on statistical inference. While there are no clear guidelines defining an acceptable proportion of missing data, the literature suggests that estimates derived using multiple imputation methods may be biased when missing data exceeds 10% (29). The maximum proportion of missing data for all variables was 7.55% (**Supplementary Table S1**); thus, the use of multiple imputation was considered appropriate. The *t*-test was used when the baseline grouped measurement data satisfied the conditions of normality and homogeneity of variances, while the Wilcoxon rank-sum test was used in cases where these criteria were not satisfied. To compare categorical variables expressed in numbers and percentages, the *X* ^2^ test or Fisher’s exact test was used.

The Running log-rank test available in the R packages “survminer” and “survival” was utilized to determine the optimal cutoff values for global strain parameters (**Supplementary Figures S1**–**S3**). Then, the Kaplan-Meier curves were generated to assess the new-onset of HF based on these determined cutoff values. To evaluate the relationship between left ventricular global strain values and the occurrence of HF, a univariate COX proportional hazards regression analysis was performed. Meanwhile, to eliminate the influence of collinearity among variables on the outcomes, a stepwise backward regression analysis was conducted on the comprehensive model encompassing all covariates to derive the ultimate model. Variables of stepwise backward regression analysis model in this study include age, sex, ethnicity, education, body mass index, systolic blood pressure, diastolic blood pressure, glycated hemoglobin, LDL-C, eGFR, drinking status, smoking status, CAD, hypertension, atrial fibrillation, diabetes medication, and diabetes duration. Stepwise regression analysis is a commonly used method which can be adopted for eliminating multicollinearity and selecting the “optimal” regression equation. The approach is to start with a model including all possible variables, with the condition that the independent variable is significant by the F test. At each step, the variables that contribute the least to model performance are selected from the current model and removed. By repeating this process, one variable is deleted at a time, until no more variables can be deleted. To sum up, the stepwise regression method we selected is ideal in screening variables and can overcome the multicollinearity of variables. Multivariate COX regression analyses with adjustment variables including age, sex, ethnicity, education, body mass index, systolic blood pressure, diastolic blood pressure, glycated hemoglobin, LDL-C, eGFR, drinking status, smoking status, CAD, hypertension, atrial fibrillation, diabetes medication, and diabetes duration were performed. This analysis was a sensitivity analysis. The predictive value of each strain indicator assessing HF was performed by the C-index and time-dependent under receiver operating characteristic curves (time-AUC). Pearson correlation analysis was utilized to determine the correlation between global strain and imaging indicators of HF. A correlation coefficient (r) between 0.1 and 0.39 is considered a weak correlation, between 0.4 and 0.69 is a moderate correlation, and between 0.7 and 0.89 is a strong correlation (30). Finally, subgroup analyses were performed to investigate the potential impact of clinical characteristics, such as age, sex, BMI, smoking status, drinking status, hypertension, and CAD, on the association between global strain and the occurrence of new-onset HF. Based on a two-sided *P*-value of ≤0.05, the statistical significance was determined.

## Results

### Population characteristics

Totally 940 patients with diabetes were included in this study. The average age of the participants was 57.67 ± 6.97 years, with males comprising 66.4% (n=624) of the overall population. With an average follow-up period of 166.82 ± 15.26 months, 35 (3.72%) patients with diabetes reached the endpoint (the emergence of new-onset HF). No patients were lost during the follow-up period. **Table 1** presents the baseline characteristics of all the participants categorized based on the presence or absence of HF during the follow-up period. The occurrence of HF during the follow-up period was significantly associated with increased age; higher BMI, systolic blood pressure, and eGFR; and the presence of CAD and hypertension. The occurrence of HF was significantly associated with decreased LVEF, increased LVESV and LVEDV, increased LVMS, and increased thickness of the myocardial wall of the left ventricle. Impaired values of LV-GRS, LV-GCS, and LV-GLS parameters are known to be associated with the occurrence of HF in individuals with diabetes.

**Table 1:** Baseline characteristics stratified by new-onset of heart failure during follow-up in patients with diabetes.

Variables	Overall	Not New-Onset Heart Failure	New-Onset Heart Failure	*P*-value
	N= 940	N=905	N=35	
Age, years	57.67 ± 6.97	57.56 ± 6.94	60.54 ± 7.29	0.013
Male, n (%)	624 (66.4)	596 (65.9)	28 (80.0)	0.120
British, n (%)	820 (87.2)	786 (86.9)	34 (97.1)	0.125
College/university degree, n (%)	378 (40.2)	367 (40.6)	11 (31.4)	0.366
Body mass index, kg/m^2^	30.38 ± 5.25	30.25 ± 5.19	33.70 ± 5.77	<0.001
Blood pressure, mmHg				
Diastolic	81.86 ± 9.05	81.78 ± 9.02	84.00 ± 9.76	0.154
Systolic	139.11 ± 15.73	138.84 ± 15.54	145.87 ± 19.14	0.009
Glycated hemoglobin, mmol/mol	50.67 ± 11.30	50.58 ± 11.29	52.97 ± 11.39	0.220
LDL-C, mmol/L	2.78 ± 0.76	2.78 ± 0.76	2.78 ± 0.74	0.999
eGFR, mL/min/1.73m2	95.48 ± 10.40	95.66 ± 10.29	90.93 ± 12.38	0.008
Drinking status, n (%)				0.673
Never	34 (3.6)	32 (3.5)	2 (5.7)	
Previous	37 (3.9)	35 (3.9)	2 (5.7)	
Current	869 (92.4)	838 (92.6)	31 (88.6)	
Smoking status, n (%)				0.382
Never	343 (36.5)	334 (36.9)	9 (25.7)	
Previous	541 (57.6)	517 (57.1)	24 (68.6)	
Current	56 (6.0)	54 (6.0)	2 (5.7)	
Coronary artery disease, n (%)	97 (10.3)	87 (9.6)	10 (28.6)	0.001
Hypertension, n (%)	577 (61.4)	546 (60.3)	31 (88.6)	0.001
Atrial fibrillation, n (%)	16 (1.7)	14 (1.5)	2 (5.7)	0.117
Diabetes medication, n (%)				0.055
Oral antidiabetic drug only	459 (48.8)	435 (48.1)	24 (68.6)	
Insulin	176 (18.7)	171 (18.9)	5 (14.3)	
Neither	305 (32.5)	299 (33.0)	6 (17.1)	
Diabetes duration, years				0.183
<1	64 (6.8)	62 (6.9)	2 (5.7)	
1-5	385 (41.0)	374 (41.3)	11 (31.4)	
5-10	309 (32.9)	299 (33.0)	10 (28.6)	
>10	182 (19.4)	170 (18.8)	12 (34.3)	
Cardiac MRI parameters				
Left ventricular ejection fraction, %	58.71 ± 6.42	58.87 ± 6.24	54.55 ± 9.35	<0.001
Left ventricular end-diastolic volume, ml	146.76 ± 32.64	145.87 ± 31.85	169.77 ± 43.54	<0.001
Left ventricular end-systolic volume, ml	61.08 ± 18.64	60.49 ± 18.05	76.33 ± 26.08	<0.001
Left ventricular myocardial mass, g	94.08 ± 21.90	93.42 ± 21.30	111.20 ± 29.54	<0.001
Left ventricular mean myocardial wall thickness, mm	6.17 ± 0.78	6.15 ± 0.78	6.61 ± 0.86	0.001
Global circumferential strain, %	-21.51 ± 3.88	-21.62 ± 3.74	-18.66 ± 5.86	<0.001
Global longitudinal strain, %	-17.78 ± 2.98	-17.85 ± 2.93	-15.87 ± 3.63	<0.001
Global radial strain, %	44.66 ± 9.04	44.85 ± 8.83	39.79 ± 12.67	0.001

Categorical variables are expressed as frequency (percentage), and continuous variables are expressed as mean (SD).

LDL-C, low density lipoprotein cholesterol; eGFR, estimated glomerular filtration rate.

### Association of global left ventricular strain with new-onset HF

**Figure 1:**
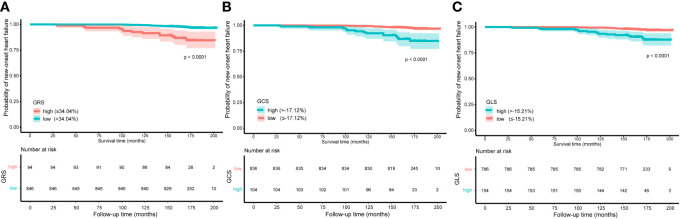
Kaplan-Meier curve of new-onset heart failure according to global radial strain **(A)**, global circumferential strain **(B)**, and global longitudinal strain **(C)** in 940 populations with diabetes. Note: patients with left ventricular global radial strain (LV-GRS) ≤ 34.04% (n= 94) had increased risk for the endpoint of new-onset heart failure (*P*-value < 0.001). Patients with left ventricular global circumferential strain (LV-GCS) > -17.12% (n= 104) had increased risk for the endpoint of new-onset heart failure (*P*-value < 0.001). Patients with left ventricular global longitudinal strain (LV-GLS) > -15.21% (n= 154) had increased risk for the endpoint of new-onset heart failure (*P*-value < 0.001). Time indicates months after cardiac magnetic resonance imaging in relation to diabetes.

**Figure 2:**
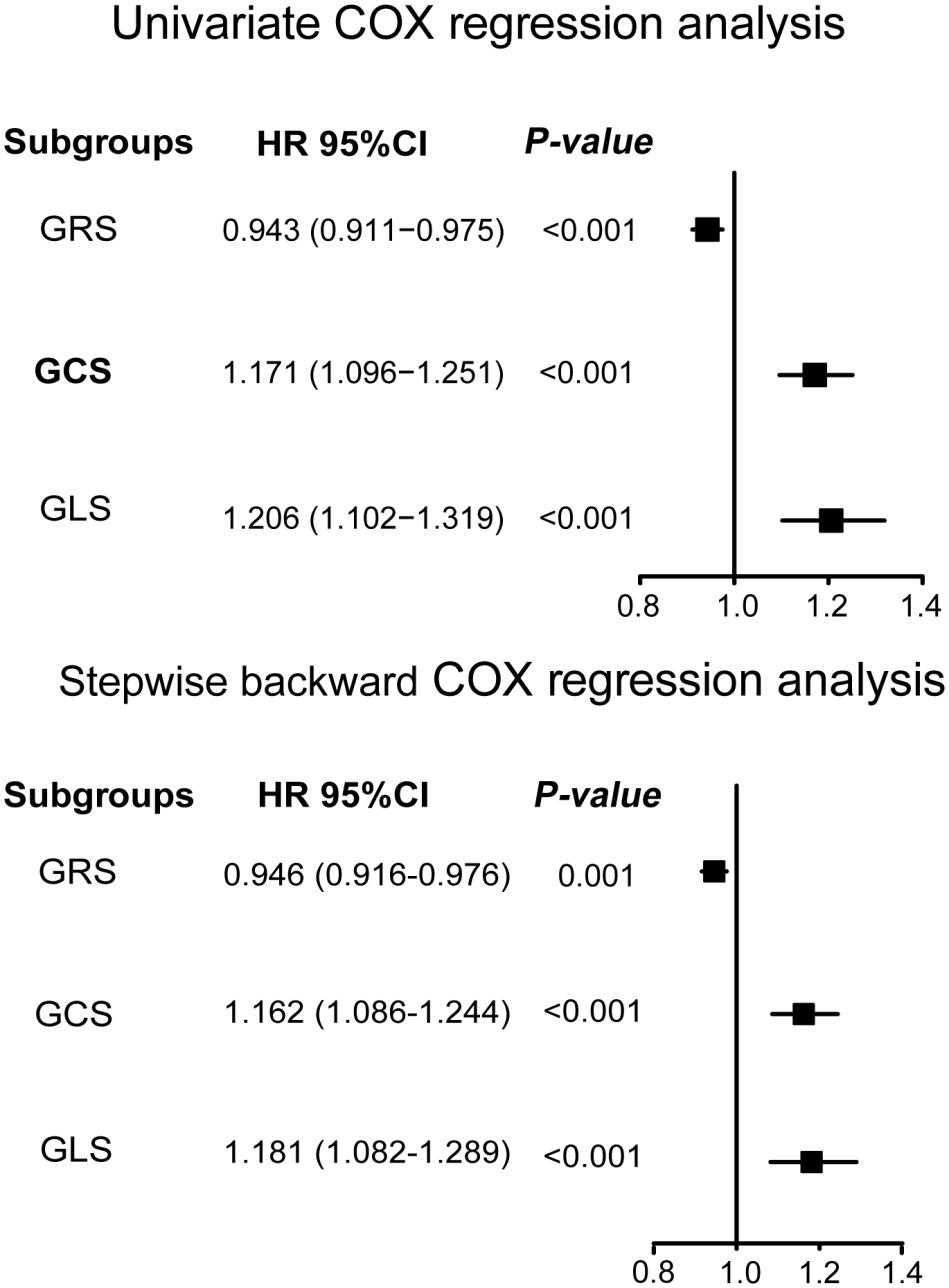
The relationship between left ventricular global strain and new-onset heart failure by univariate and stepwise backward regression analysis. Variables of stepwise backward regression analysis model in this study include age, sex, ethnicity, education, body mass index, systolic blood pressure, diastolic blood pressure, glycated hemoglobin, LDL-C, eGFR, drinking status, smoking status, coronary artery disease, hypertension, atrial fibrillation, diabetes medication, diabetes duration, LV-GRS, LV-GCS, and LV-GLS. LV-GRS, left ventricular global radial strain; LV-GCS, left ventricular global circumferential strain; LV-GLS, left ventricular global longitudinal strain; LDL-C, low density lipoprotein cholesterol; eGFR, estimated glomerular filtration rate.

The Running log-rank test was used to determine the optimal cutoff values for LV-GRS, LV-GCS, and LV-GLS, respectively. When comparing participants with LV-GCS ≤–17.12%, it was found that a cutoff value of LV-GCS >–17.12% (HR: 5.64; 95% CI: 2.87 to 11.09; *P <*0.001) identified 104 individuals with diabetes who were at a high risk of the emergence of new-onset HF. Similarly, individuals with LV-GLS ≤–15.21% were compared, and a cutoff value of LV-GLS >–15.21% (HR: 4.50; 95% CI: 2.31 to 8.75; *P <*0.001) identified 154 high-risk individuals with diabetes. By contrast, individuals who had diabetes with LV-GRS ≤34.04% were compared, and a cutoff value of LV-GRS >34.04% (HR: 0.18; 95% CI: 0.09 to 0.35; *P <*0.001) identified 846 individuals at a low risk for developing diabetes. The Kaplan-Meier estimate dichotomized according to LV-GRS ≤34.04%, LV-GCS ≤-17.12%, and LV-GLS ≤-15.21% is presented in **Figure 1** (log-rank chi-square 31.54, *P <*0.0001; log-rank chi-square 32.05 (*P <*0.0001), and log-rank chi-square 23.64 (*P <*0.0001), respectively). A time-AUC and C-index were performed to exhibit the prognostic ability of global strain parameters in patients with diabetes. The results of the univariate and multivariate COX regression analyses are illustrated in **Figure 2**. The univariate COX regression analysis, representing a crude model, revealed statistically significant associations between a decrease in LV-GRS, an increase in LV-GCS, and an increase in LV-GLS with increased hazards of developing HF in the individuals suffering from diabetes. Similar results were observed in the backward stepwise regression model (**Figure 2**; **Supplementary Table S2**). Similarly, in sensitivity analyses, baseline variables were adjusted in multivariate COX regressions with results consistent with the main study (**Supplementary Table S3**).

### Prognostic value of global left ventricular strain for new-onset HF

**Figure 3:**
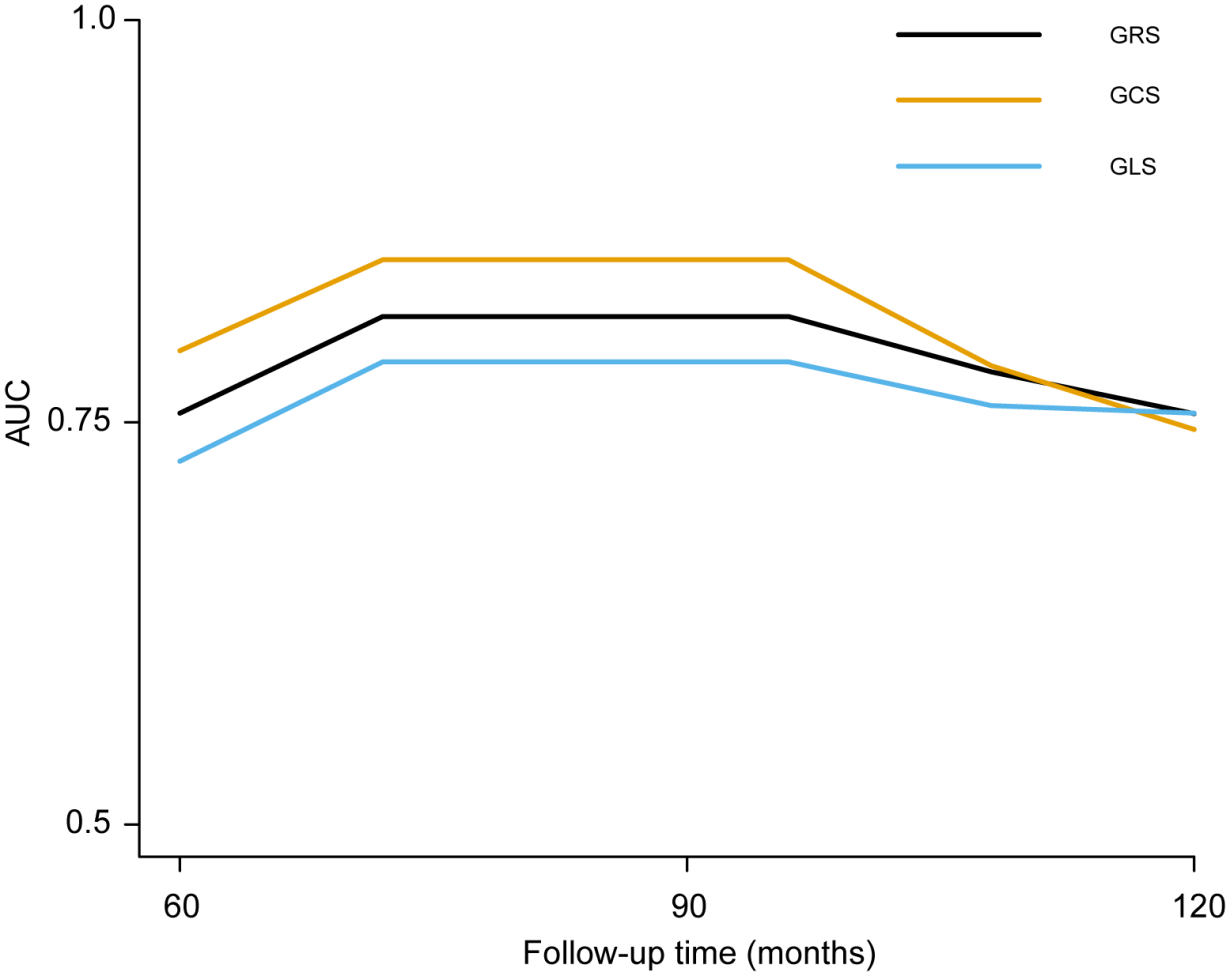
The time‐dependent AUC of left ventricular global strain for predicting new-onset HF in patients with diabetes. LV-GRS, left ventricular global radial strain; LV-GCS, left ventricular global circumferential strain; LV-GLS: left ventricular global longitudinal strain; HF, heart failure. LV-GRS, left ventricular global radial strain; LV-GCS, left ventricular global circumferential strain; LV-GLS, left ventricular global longitudinal strain.

As displayed in **Figure 3** and **Table 2**, LV-GRS showed the C-index for new-onset HF in participants with diabetes at 5 and 10 years: 0.75 (95% CI, 0.41-0.94) and 0.76 (95% CI, 0.44-0.98), respectively. LV-GCS showed the C-index for new-onset HF in participants with diabetes at 5 and 10 years: 0.80 (95% CI, 0.50-0.96) and 0.75 (95% CI, 0.41-0.98), respectively. LV-GLS exhibited the C-index for new-onset HF in participants with diabetes at 5 and 10 years: 0.72 (95% CI, 0.40-0.93) and 0.76 (95% CI, 0.48-0.97), respectively. To compare the predictive value of LVEF, LV-GRS, LV-GCS and LV-GLS in predicting new-onset HF among patients with diabetes, ROC curves and related parameters were provided in **Supplementary Figure S4** and **Supplementary Table S4**. The AUC of LV-GCS and LV-GLS for predicting new-onset HF in diabetic patients was slightly higher than LVEF. The AUC of LV-GRS was slightly lower than LVEF (The AUC of LVEF, LV-GRS, LV-GCS and LV-GLS: 0.643, 0.624, 0.663, and 0.668, respectively).

**Table 2:** The C-index of left ventricular global strain for new-onset HF in patients with diabetes.

Indicators	*C*‐index (95% CI)	
	5‐year	10‐year
LV-GRS	0.75 (0.41, 0.94)	0.76 (0.44, 0.98)
LV-GCS	0.80 (0.50, 0.96)	0.75 (0.41, 0.98)
LV-GLS	0.72 (0.40, 0.93)	0.76 (0.48, 0.97)

LV-GRS, left ventricular global radial strain; LV-GCS, left ventricular global circumferential strain; LV-GLS, left ventricular global longitudinal strain.

### Correlation between global left ventricular strain and imaging indicators related to HF

In the cohort of individuals with newly diagnosed HF, LV-GCS and LV-GLS exhibited a significant negative correlation with LVEF (LV-GCS: *r* = –0.85, *P*  <0.001 and LV-GLS: *r* = –0.80, *P*  < 0.001), while displaying a positive correlation with LVEDV (LV-GCS: *r* = 0.49, *P*=0.003 and LV-GLS: *r* = 0.39, *P*=0.020), LVESV (LV-GCS: *r* = 0.76, *P <*0.001 and LV-GLS: *r* = 0.62, *P <*0.001), LVMS (LV-GCS: *r* = 0.58, *P <*0.001 and LV-GLS: *r* = 0.55, *P <*0.001), and left ventricular mean myocardial wall thickness global (LV-GCS: *r* = 0.42, *P*=0.010 and LV-GLS: *r* = 0.51, *P*=0.002). By contrast, LV-GRS demonstrated a positive correlation with LVEF (*r* = 0.81, *P* < 0.001) and a negative correlation with LVEDV (*r* =–0.41, *P*=0.020), LVESV (*r* =–0.64, *P <*0.001), LVMS (*r* =–0.53, *P*=0.001), and left ventricular myocardial wall thickness (*r* = –0.39, *P*=0.020). Similar findings were observed in the group of individuals without newly diagnosed HF (**Table 3**).

**Table 3:** Correlation between left ventricular global strain and heart failure related indicators by the presence or absence of new-onset heart failure.

Variables	LV-GRS			
	Not New-Onset Heart Failure		New-Onset Heart Failure	
	r	*P*-value	r	*P*-value
Left ventricular ejection fraction	0.67	<0.001	0.81	<0.001
Left ventricular myocardial mass	-0.27	<0.001	-0.53	0.001
Left ventricular end-diastolic volume	-0.30	<0.001	-0.41	0.020
Left ventricular end-systolic volume	-0.54	<0.001	-0.64	<0.001
Left ventricular mean myocardial wall thickness	-0.11	<0.001	-0.39	0.020
Variables	LV-GCS			
	Not New-Onset Heart Failure		New-Onset Heart Failure	
	r	*P*-value	r	*P*-value
Left ventricular ejection fraction	-0.75	<0.001	-0.85	<0.001
Left ventricular myocardial mass	0.31	<0.001	0.58	<0.001
Left ventricular end-diastolic volume	0.34	<0.001	0.49	0.003
Left ventricular end-systolic volume	0.62	<0.001	0.76	<0.001
Left ventricular mean myocardial wall thickness	0.18	<0.001	0.42	0.010
Variables	LV-GLS			
	Not New-Onset Heart Failure		New-Onset Heart Failure	
	r	*P*-value	r	*P*-value
Left ventricular ejection fraction	-0.42	<0.001	-0.80	<0.001
Left ventricular myocardial mass	0.21	<0.001	0.55	<0.001
Left ventricular end-diastolic volume	0.009	0.78	0.39	0.020
Left ventricular end-systolic volume	0.21	<0.001	0.62	<0.001
Left ventricular mean myocardial wall thickness	0.29	<0.001	0.51	0.002

LV-GRS, left ventricular global radial strain; LV-GCS, left ventricular global circumferential strain; LV-GLS, left ventricular global longitudinal strain.

### Subgroup analysis

**Figure 4:**
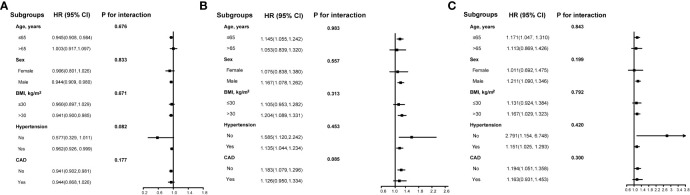
The subgroups analyses of the relationship between left ventricular global strain and the incidence of new-onset heart failure in populations with diabetes. **(A)** left ventricular global radial strain. **(B)** left ventricular global circumferential strain. **(C)** left ventricular global longitudinal strain. Adjusted variables include age, sex, ethnicity, education, body mass index, systolic blood pressure, diastolic blood pressure, glycated hemoglobin, LDL-C, eGFR, drinking status, smoking status, coronary artery disease, hypertension, atrial fibrillation, diabetes medication, and diabetes duration. LV-GRS, left ventricular global radial strain; LV-GCS, left ventricular global circumferential strain; LV-GLS, left ventricular global longitudinal strain; LDL-C, low density lipoprotein cholesterol; eGFR, estimated glomerular filtration rate.

Subgroup analyses were conducted to assess the correlation between left ventricular global strain parameters (LV-GRS, LV-GCS, and LV-GLS) and the occurrence of HF based on various factors including age, sex, BMI, and the presence of hypertension or CAD (**Figure 4**). After the adjustment for confounders, no substantial interaction was detected between the subgroup variables in the association between GRS, GCS, and GLS and the occurrence of HF (*P*>0.05).

## Discussion

This study is a large prospective cohort study showing that lower LV-GRS and higher LV-GCS and LV-GLS were independently correlated with higher risk of HF in a diabetic population. A running log-rank test was used to determine the optimal cut-off values for LV-GRS, LV-GCS, and LV-GLS in assessing new-onset HF in diabetic patients, which were 34.04%, -17.12%, and -15.21%, respectively. Moreover, this helped us to identify subgroups of diabetic population at high risk of heart failure. In participants with subsequent HF, baseline LV-GRS, LV-GCS, and LV-GLS levels were strongly correlated with LVEF and moderately related to left ventricular myocardial mass and end-systolic volume. Additionally, LV-GRS, LV-GCS, and LV-GLS had good predictive value for developing heart failure in diabetic patients after 5 and 10 years. Particularly, LV-GLS and LV-GCS predicted HF slightly better than traditional LVEF indices. Age, sex, BMI, and the presence of hypertension or CAD made no impact on the relationship between global strain parameters and HF incidence.

Patients with diabetes are at a higher risk of developing HF following a cardiac event compared with those without diabetes. This increased susceptibility to HF may be caused by subclinical LV dysfunction, and multiple studies have demonstrated disruptions in nuanced indicators of systolic and diastolic function using tissue Doppler and strain and strain rate imaging (20, 31, 32). Thais et al. summarized totally 27 studies, discovering that using 2D and 3D speckle-tracking echocardiography for measuring myocardial strain effectively detected subclinical systolic dysfunction among patients diagnosed with diabetes (33). However, their study was limited to studies on cardiomyopathy caused by diabetes, and only compared the differences in strain values between the cardiomyopathy group and the non-myocardial group. It is well-known that cardiac dysfunction in some patients with diabetes cannot be entirely attributed to diabetic cardiomyopathy (34). Obesity and the presence of hypertension or CHD exert a substantial impact on the occurrence of HF (35–37). Therefore, in this study, we comprehensively considered various influencing factors that might contribute to the development of HF and found that left ventricular global strain (LV-GRS, LV-GCS, and LV-GLS) exhibited a good diagnostic ability in assessing the occurrence of HF in individuals with diabetes. Shu et al. employed cardiac magnetic resonance-derived LV-GCS to evaluate the prognostic value of DCM patients with severely reduced ejection (clinical composite endpoint: all-cause mortality, cardiac transplantation, implantable cardioverter-defibrillator implantation, and sudden cardiac death), finding that compared with patients with LV-GCS < -5.17%, patients with LV-GCS ≥ -5.17% had a significantly lower event-free survival rate (*P*<0.01) (38). This is in consistence with our findings. In this study, using Running log-rank test to identify the optimal cutoff values of LV-GRS, LV-GCS, and LV-GLS for assessing new-onset heart failure in diabetic patients (LV-GRS: 34.0387%; LV-GCS: -17.1177%; LV-GLS: -15.2089%), it could be found that the values of LV-GRS, LV-GCS and LV-GLS in the diabetic population without heart failure were significantly higher than those in the diabetic population with heart failure. In addition, Tröbset al. found that each unit increase in echocardiography-derived LV-GLS was associated with a 55% increase in all-cause mortality (*P* <.001) and a 132% increase in cardiac death (*P* < 0.001) in patients suffering from chronic heart failure (39). In this study, we investigated the relationship between CMR-derived LV-GRS, LV-GCS, and LV-GLS and heart failure in diabetic patients and indicated that the incidence of heart failure in diabetic patients decreased by 5.4% for every 1% increase in LV-GRS, for every 1% increase in LV-GCS, the incidence of heart failure in diabetic patients increased by 16.2%, and for every 1% increase in LV-GLS, the incidence of heart failure in diabetic patients increased by 18.1%.

Left ventricular strain, especially as measured by CMRI, has been shown to exhibit potential prognostic value for the assessment of cardiovascular disease. Wang et al. discovered that the strain parameters derived from CMRI, specifically LV-GLS, LV-GRS, LV-GCS, and left atrial strain (LAS), are potential diagnostic markers for accurately distinguishing between light-chain cardiac amyloidosis and hypertrophic cardiomyopathy (40). Cao et al. found that cardiac magnetic resonance tissue tracking could effectively detect early biventricular myocardial damage in patients with pulmonary hypertension and shows good feasibility and reproducibility (41). Similarly, Zhou et al. discovered that early left ventricular systolic and diastolic function in prediabetic and diabetic groups could be reflected by parameters derived from cardiovascular magnetic resonance-feature tracking (42). Despite numerous studies, whether strain parameters derived from CMRI, markers of impaired myocardial systolic or diastolic function, are related to the development of HF in individuals with diabetes has not yet been explored. Therefore, this study included 940 individuals with diabetes from the UK biobank database undergoing CMRI. The results revealed that LV-GRS, LV-GCS, and LV-GLS were all independently associated with the occurrence of HF in patients with diabetes [LV-GRS: HR: 0.946 (95%CI: 0.916-0.976); LV-GCS: HR: 1.162 (95%CI: 1.086-1.244); LV-GLS: HR: 1.181 (95%CI: 1.082-1.289)]. Age, sex, BMI, and the presence of hypertension or CAD made no impact on the relationship between left ventricular global strain parameters (LV-GRS, LV-GCS, and LV-GLS) and HF. The above results show that LV-RS, LV-CS and LV-LS are independently related to the occurrence of heart failure in diabetic patients. Park et al. found that for every 1% increase in GLS, the risk of mortality increased by 5% in patients with acute heart failure (*P* < 0.001), and LVEF was not associated with mortality, concluding that GLS has greater prognostic value than LVEF in patients with acute heart failure (43). Similarly, we found that LV-GLS and LV-GCS may be slightly better than LVEF in predicting heart failure findings in patients with diabetes [LV-GCS: AUC=0.663; LV-GLS: AUC=0.668; LVEF: AUC=0.643].

The myocardial velocities include longitudinal, radial, circumferential, and spiral. As the myocardium moves in different directions, the value of the strain changes accordingly. LV-GLS has been demonstrated to be a reproducible and valuable tool for elucidating the association between HF and mortality (44). A higher degree of impaired LV-GLS serves as a robust and autonomous prognosticator for long-term all‐cause mortality, as well as HF admissions, among African American individuals diagnosed with chronic stable HF and receiving optimal medical therapy per the established guidelines (45). Vietheer et al. indicated that ischemic and dilated cardiomyopathy could be effectively distinguished based on the severity of impaired LV-GCS (not LV-GLS and not LV-GRS) in CMRI (46). Shu et al. showed that LV-GCS derived from CMRI could be beneficial for risk stratification in patients with dilated cardiomyopathy with severely reduced EF (38). Sardana et al. found that LV-GRS, LV-GCS, and LV-GLS were independently associated with adverse outcomes in participants with a preserved LVEF (47). LV-GRS and average  LV-GLS were values the only CMR parameters to be significantly correlated with 5-year all-cause mortality in patients with HF (48). However, there are no consistent reports on the diagnostic value of LV-GRS, LV-GCS, and LV-GLS derived from CMRI for assessing cardiovascular disease. This inconsistency in results may be because of the small sample size and short follow-up time of each study. In this study, totally 940 patients with diabetes with no signs of HF were included and followed up for an average of 166.82 ± 15.26 months. Among them, 35 (3.72%) patients with diabetes developed HF. 100% follow-up rate and long follow-up time could ensure the reliability of the results. Ernande et al. followed 172 asymptomatic patients with type 2 diabetes for 3 years, finding that longitudinal strain was closely associated with closely related indicators of left ventricular remodeling (LVEDV: β = 19.1, P =0.001; LVESV: β = 2.6, P =0.047) (49). Consistently, we found that LV-GRS, LV-GCS, and LV-GLS derived from CMRI tissue tagging exhibited a close correlation with the related indicators to HF and could be adopted for risk stratification in patients with diabetes. When Liu et al. investigated the relationship between LV-GLS and cardiovascular events in patients with diabetes (cardiovascular events: acute coronary syndrome, cerebrovascular stroke, heart failure hospitalization, and cardiovascular death), they found that GLS > -17.9% was correlated with cardiovascular events, and LV-GLS was a strong predictor of cardiovascular events (AUC 0.72) (50). Different from their study, we simultaneously explored the relationship between LV-GRS, LV-GCS and LV-GLS and the occurrence of heart failure in patients with diabetes, discovering that impaired LV-GRS, LV-GCS, and LV-GLS values had a high relationship to the occurrence of HF at 5 and 10 years.

## Limitations

The occurrence of events among participants was relatively low (n=940/35,896, 3.72%), thereby limiting our ability to identify a statistically significant correlation between overall strain and event incidence as well as impeding further investigation into the association between strain and different types of HF. Owing to the small proportion of participants who experienced events, the study population with diabetes was not further divided into type I and type II diabetes, highlighting the need for a larger sample size in future research endeavors. Finally, concerning the observational nature of the study, it was imperative to acknowledge the potential presence of residual confounding or reverse causation. Nevertheless, it is vital to emphasize that the primary aim of this study was to provide a descriptive analysis of the associations rather than establishing causal inferences. This was an observational study, limited to the collection of baseline data. As a result, we were unable to predict or assess the potential impact of certain diseases, including COVID-19, that occurred during the follow-up period. These events may have a significant impact on the results of the study, so the effect of these diseases on our results is uncertain. In addition, obtaining baseline medical information by self-reports may introduce memory bias and affect study results. We analyzed three indices—LV-GRS, LV-GCS, and LV-GLS—due to their comprehensive assessment of cardiac function and myocardial deformability. Other strain parameters, such as 3D strain and strain rate, were less utilized in daily clinical practice and not included in the UK Biobank dataset, primarily due to technical limitations, costs, and measurement complexity. Furthermore, N-terminal B-type natriuretic peptide (NT-pro BNP) and high-sensitivity cardiac troponin T (HS-cTNT) serve as highly sensitive markers for cardiac injury, predictive of heart failure and other cardiovascular diseases. Even baseline levels within the normal range of NT-pro BNP and HS-cTNT have been linked to an increased risk of subsequent heart failure. However, our study, enrolling participants from 2006 to 2010, could not incorporate NT-pro BNP and HS-cTNT data for analysis, as assays for these markers were not widely used in the UK Biobank data collection at that time.

### Clinical implications

In clinical practice, CMR imaging is typically used for specific conditions requiring detailed cardiac evaluation; it is not routinely performed and is particularly rare in general population screening. The current study provides the first in-depth analysis of the association of LV global strain indices - LV-GRS, LV-GCS, and LV-GLS - with the subsequent occurrence of HF in diabetic patients. We observed that lower LV-GRS, and higher LV-GCS and LV-GLS were independently associated with an increased risk of HF. Using the log-rank test, optimal thresholds for these strain indicators were determined, providing an important basis for identifying diabetic patients at high risk of HF. In addition, these strain indicators have good predictive value for the onset of HF in diabetic patients over the next 5 to 10 years. In particular, LV-GLS and LV-GCS were slightly better at predicting heart failure than conventional LVEF. These results suggest that LV global strain has the potential to become a useful risk assessment tool for diabetic patients.

Early identification of individuals at high risk of HF in diabetic patients, preventive measures and personalized management are essential. CMR imaging provides a comprehensive evaluation of cardiac structure and myocardial deformation function, and is able to capture signs of subclinical cardiac damage that are not readily observable with cardiac ultrasound. Early identification of these injuries allows physicians to take more appropriate therapeutic measures, such as adjusting treatment regimens, recommending lifestyle changes, or performing regular cardiac status monitoring to prevent the progression of HF. CMR imaging serves not just for diagnosis but also as a foundation for crafting personalized treatment plans to lower HF risk.

Currently, the use of CMR in diabetic patients is not widely available, but its potential for comprehensive assessment of myocardial function is promising. Integration of CMR into the diabetes management process would enable more accurate risk stratification. Therefore, early consideration of CMR use in the management of diabetic patients is encouraged. This approach helps to identify subtle changes in cardiac function in a timely manner, optimizing the patient’s treatment strategy and prognosis.

## Conclusion

LV-GRS, LV-GCS, and LV-GLS are significantly related to new-onset HF in patients with diabetes at 5 and 10 years.

## Data availability statement

The datasets presented in this study can be found in online repositories. The names of the repository/repositories and accession number(s) can be found in the article/**Supplementary Material**.

## Ethics statement

The studies involving humans were approved by North West Multi-Centre Research Ethics Committee. The studies were conducted in accordance with the local legislation and institutional requirements. The participants provided their written informed consent to participate in this study.

## Author contributions

SC: Writing – review & editing, Writing – original draft, Validation, Supervision, Software, Formal analysis, Data curation, Conceptualization. CC: Writing – review & editing, Writing – original draft, Conceptualization. LZ: Writing – review & editing, Writing – original draft, Software, Investigation, Data curation. WC: Writing – review & editing, Writing – original draft, Supervision, Resources, Formal analysis. XB: Writing – review & editing, Writing – original draft, Investigation. ZL: Writing – review & editing, Writing – original draft, Visualization, Supervision, Funding acquisition, Formal analysis, Data curation.
