# Supplementary material for: Assessment of new-onset heart failure prediction in a diabetic population using left ventricular global strain: a prospective cohort study based on UK Biobank
Source: Front Endocrinol (Lausanne). 2024 Apr 2;15:1365169. doi: 10.3389/fendo.2024.1365169 (PMC11018882; doi:10.3389/fendo.2024.1365169)
Supplement: Supplementary file 2 [file DataSheet_2.pdf]

Supplement

Assessment of New-Onset Heart Failure Prediction in a Diabetic Population Using Global Left Ventricular Strain: A Prospective Cohort Study Based on UK Biobank

Siwei Chen, Cong Chen, Longxuan Zheng, Wenke Cheng, Xiancong Bu, Zhou Liu

Figure S1 Determination of optimal cutoff value for GRS on assessing development of HF during to following-up.
Figure S2 Determination of optimal cutoff value for GCS on assessing development of HF during to following-up.
Figure S3 Determination of optimal cutoff value for GLS on assessing development of HF during to following-up.
Figure S4 The ROC curves of LVEF, LV-GRS, LV-GCS and LV-GLS for predicting new-onset heart failure among patients with diabetes.
Table S1 The proportions and numbers of missing baseline data.
Table S2 Stepwise backward regression analyses for global strain on assessing development of HF during to following-up.
Table S3 Multivariate COX regression analysis for left ventricular global strain on assessing development of HF during to following-up.
Table S4. The AUC of LVEF, LV-GRS, LV-GCS and LV-GLS for predicting new-onset heart failure among patients with diabetes.
Appendix 1 Code for R software and Stata software

Figure S1 Determination of optimal cutoff value for GRS on assessing development of HF during to following-up.

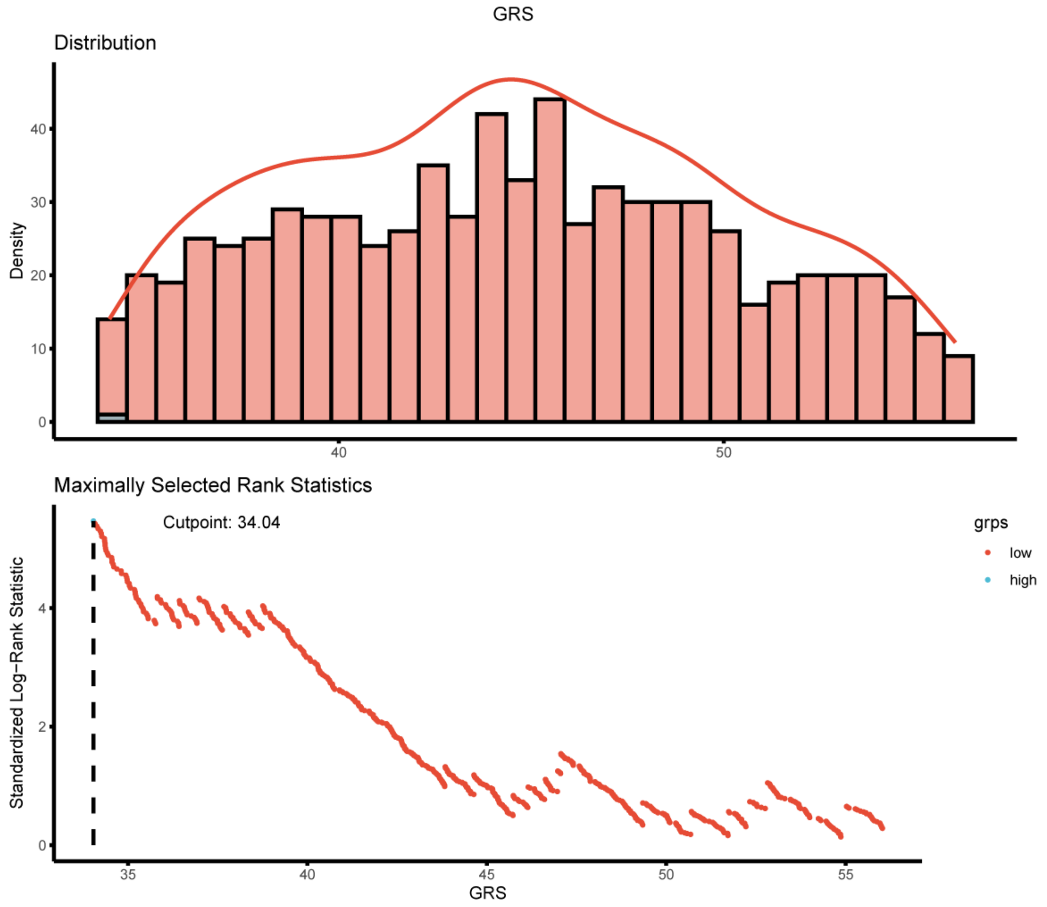

Figure S2 Determination of optimal cutoff value for GCS on assessing development of HF during to following-up.

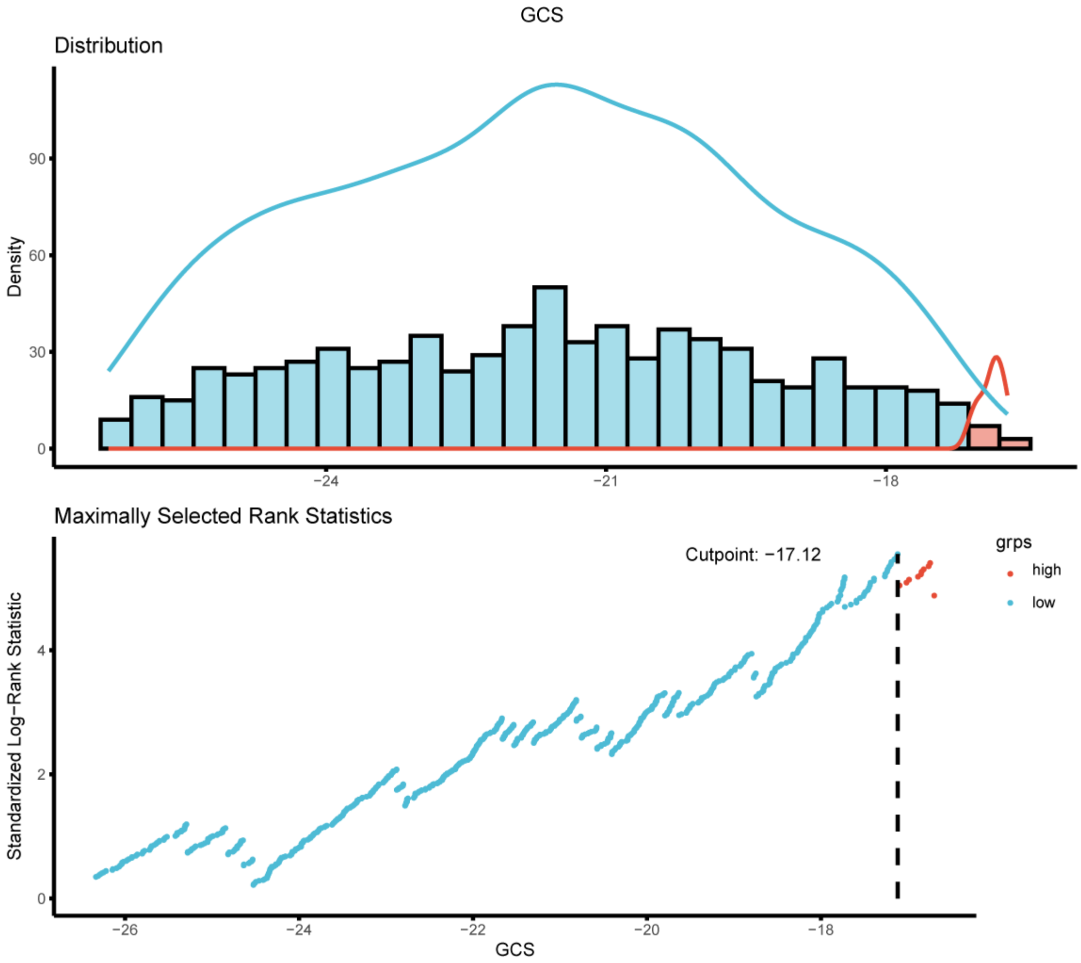

Figure S3 Determination of optimal cutoff value for GLS on assessing development of HF during to following-up.

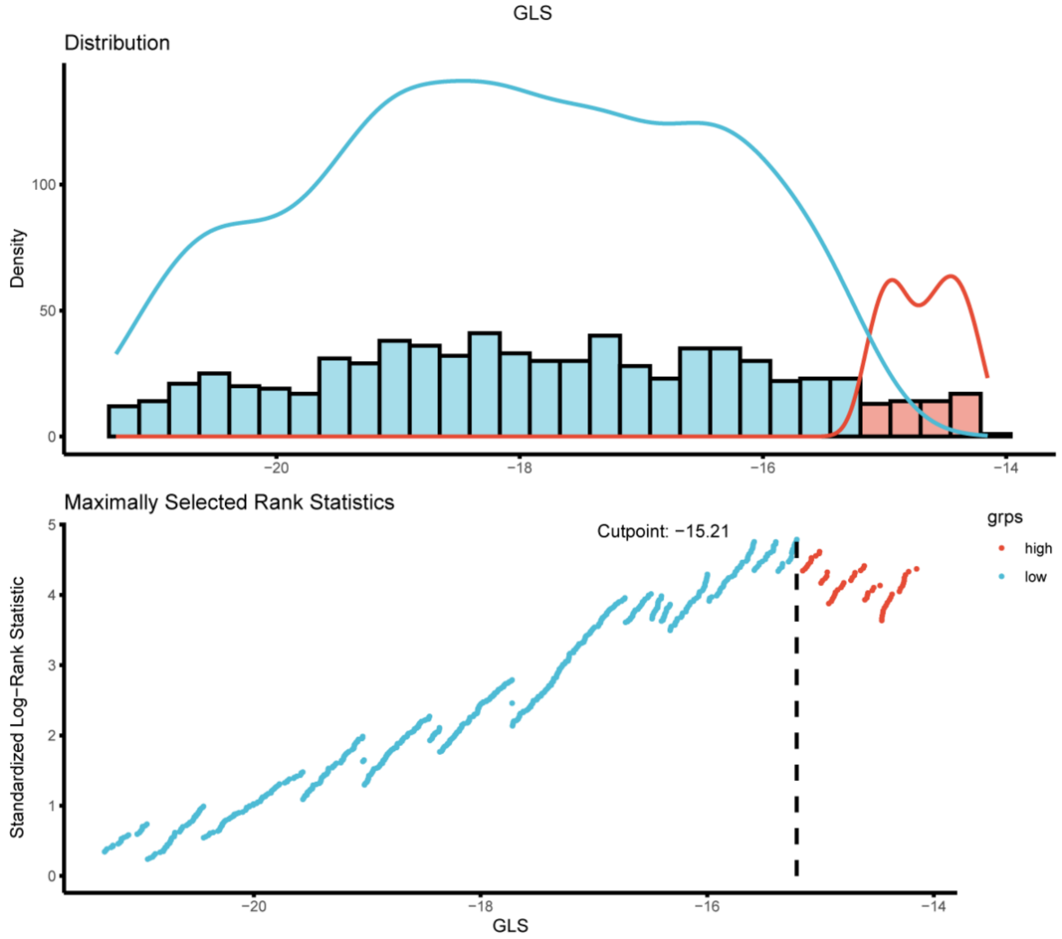

Figure S4 The ROC curves of LVEF, LV-GRS, LV-GCS and LV-GLS for predicting new-onset heart failure among patients with diabetes.

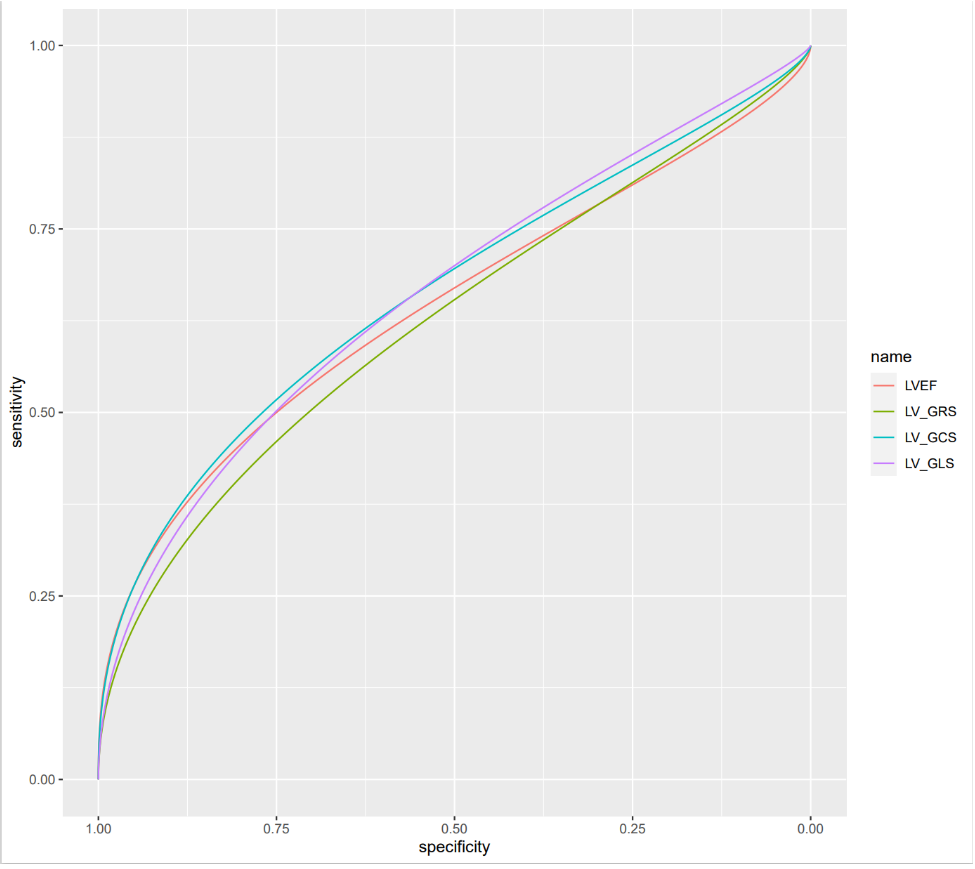

Table S1 The proportions and numbers of missing baseline data.

Variables	Number of missing values	Proportion of missing values
Age, years	0	0%
Sex, n (%)	0	0%
British, n (%)	1	0.11%
College/university degree, n (%)	13	1.38%
Body mass index, kg/m^2	2	0.21%
Diastolic blood pressure, mmHg	1	0.11%
Systolic blood pressure, mmHg	1	0.11%
eGFR, mL/min/1.73m2	70	7.45%
Glycated hemoglobin, mmol/mol	69	7.34%
LDL-C, mmol/L	71	7.55%
Drinking status, n (%)	0	0.21%
Smoking status, n (%)	0	0.53%
Coronary heart disease, n (%)	0	0%
hypertension, n (%)	0	0%
Diabetes duration, years	0	0%
**Diabetes medication, n (%)**	0	0%
Left ventricular ejection fraction, %	0	0%
Left ventricular end-diastolic volume, ml	0	0%
Left ventricular end-systolic volume, ml	0	0%
Left ventricular myocardial mass, g	0	0%
Left ventricular mean myocardial wall thickness, mm	0	0%
GCS	0	0%
GLS	0	0%
GRS	0	0%
New heart failure	0	0%

Table S2 Stepwise backward regression analyses for global strain on assessing development of HF during to following-up.

Variables	GRS[a]		GCS[b]		GLS[c]	
	HR (95% CI)	*P*-value	HR (95% CI)	*P*-value	HR (95% CI)	*P*-value
Age	1.078 (1.017-1.142)	0.011			1.070 (1.010-1.134)	0.022
Male						
British						
College/university degree						
Body mass index	1.118 (1.051-1.189)	<0.001	1.108 (1.042-1.178)	0.001	1.126 (1.059-1.198)	<0.001
Diastolic blood pressure						
Systolic blood pressure			1.024 (1.004-1.045)	0.017	1.025 (1.004-1.045)	0.018
Glycated hemoglobin						
LDL-C						
Drinking status						
Smoking status						
Coronary artery disease	2.127 (1.009-4.486)	0.047	2.206 (1.026-4.744)	0.043	3.018 (1.429-6.374)	0.004
hypertension	3.425 (1.186-9.897)	0.023	3.005 (1.019-8.864)	0.046		
Atrial fibrillation						
Diabetes medication						
Diabetes duration						
eGFR			0.964 (0.935-0.995)	0.023		
LV-GCS			1.162 (1.086-1.244)	<0.001		
LV-GLS					1.181 (1.082-1.289)	<0.001
LV-GRS	0.946 (0.916-0.976)	0.001				

Abbreviation: LV-GRS, left ventricular global radial strain; LV-GCS, left ventricular global circumferential strain; LV-GLS, left ventricular global longitudinal strain; LDL-C, low density lipoprotein cholesterol; eGFR, estimated glomerular filtration rate.

[a] Indicates that the backward stepwise regression model eventually adjusted for age, body mass index, coronary artery disease, and hypertension.

[b] Indicates that the backward stepwise regression model eventually adjusted for body mass index, systolic blood pressure, coronary artery disease, hypertension and eGFR.

[c] Indicates that the backward stepwise regression model eventually adjusted for age, body mass index, systolic blood pressure, and coronary artery disease.

Table S3 Multivariate COX regression analysis for left ventricular global strain on assessing development of HF during to following-up.

Variables	HR (95% CI)	*P*-value
LV-GRS	0.948 (0.915-0.982)	0.003
LV-GCS	1.155 (1.072-1.245)	<0.001
LV-GLS	1.170 (1.058-1.293)	0.002

Abbreviation: LV-GRS, left ventricular global radial strain; LV-GCS, left ventricular global circumferential strain; LV-GLS, left ventricular global longitudinal strain; LDL-C, low density lipoprotein cholesterol; eGFR, estimated glomerular filtration rate.

Variables were adjusted in the multiple COX regression including age, sex, ethnicity, education, body mass index, systolic blood pressure, diastolic blood pressure, glycated hemoglobin, LDL-C, eGFR, drinking status, smoking status, coronary heart disease, hypertension, atrial fibrillation, diabetes medication, and diabetes duration.

Table S4. The AUC of LVEF, LV-GRS, LV-GCS and LV-GLS for predicting new-onset heart failure among patients with diabetes.

Variables	AUC	95% CI
LVEF	0.643	0.611 - 0.673
LV-GRS	0.624	0.592 - 0.655
LV-GCS	0.663	0.632 - 0.693
LV-GLS	0.668	0.637 - 0.698

Abbreviation: LV-GRS, left ventricular global radial strain; LV-GCS, left ventricular global circumferential strain; LV-GLS, left ventricular global longitudinal strain; LDL-C, low density lipoprotein cholesterol; eGFR, estimated glomerular filtration rate.

R Code

```
library(riskRegression)
set.seed(9)
library(survival)
library(prodlim)
library(foreign)
library(forestplot)
library(survminer) # Load survminer package
library(survival) # Load survival package
library(ggplot2)
library(magrittr)
library(nhanesR)
library(ggstatsplot)
library(plyr)
library(haven)

#### View all variable names (optional step for easy copy-paste of variable names)
colnames(data)

dput(names(data))
## Convert categorical variables to factor variables in batch
# Categorical variables
factorCols <- c("sex", "eth", "edu", "smoke", "drink", "chf", "cad",
                "hypertension", "hyperlipidemia", "dm",
                "ckd","stroke", "status")
# Convert to factors in batch
for(i in factorCols) {data[,i] <- as.factor(data[,i])}

unusual_num <- function(colname, df) {
  # Missing values
  na_num <- length(which(is.na(data[[colname]])))
  na_rate <- paste(round(na_num / length(data[[colname]]), 4)*100, "%" , sep = '')
  if(class(data[[colname]]) == "factor") {
    result <- data.frame('Variable Name'=colname, 'Lower Bound' = '-', 'Upper Bound' = '-', 'Number of Outliers' = '-', 'Number of Missing Values' = na_num, 'Missing Rate' = na_rate)
    return (result)
  }
  s <- summary(data[[colname]])
  # Calculate lower bound l=Q1－1.5*IQR
  IQR <- as.numeric(s[5] - s[2])
  l <- round(as.numeric(s[2] - 1.5*IQR),3)
  # Calculate upper bound u=Q3+1.5*IQR
  u <- round(as.numeric(s[5] + 1.5*IQR),3)
  unusual_num <- length(which((df[[colname]]<(l)|(df[[colname]]>(u)))))
  result <- data.frame('Variable Name'=colname, 'Lower Bound' = l, 'Upper Bound' = u, 'Number of Outliers' = unusual_num, 'Number of Missing Values' = na_num, 'Missing Rate' = na_rate)
  return (result)
}

######## Multiple Imputation
```

```
# Use the mice package for multiple imputation
library(mice)

# Define mode function, copy and paste directly
getmode <- function(v) {
  uniqv <- unique(v)
  uniqv[which.max(tabulate(match(v, uniqv)))]
}
# Define function to fill missing values, using pmm method here.
fix_na <- function(factorCols, data, n) {
  imp = mice(data, m = n, method = "pmm")
  datas = list()
  for(i in c(1:n)) datas[[i]] <- complete(imp, action = i)
  colnames <- c()
  numeric_cols <- c()
  result <- 1 : length(datas[[1]][,1])
  allCols <- colnames(datas[[1]])
  for(colname in allCols) {
    temp <- 1 : length(datas[[1]][,1])
    for(data in datas) temp <- cbind(temp, data[[colname]])
    temp <- temp[, -1]
    if(colname %in% factorCols) {
      mode_value <- apply(temp, 1, getmode)
      result <- cbind(result, mode_value)
    } else {
      mean_value <- apply(temp, 1, mean)
      result <- cbind(result, mean_value)
      numeric_cols <- c(numeric_cols, colname)
    }
    colnames <- c(colnames, colname)
  }
  result <- as.data.frame(result[, -1])
  names(result) <- colnames
  for(i in factorCols) result[[i]] = as.factor(result[[i]])
  for(i in numeric_cols) result[[i]] = as.numeric(result[[i]])
  return (result)
}

## Call function to fill missing values

dput(names(data))
# Define factor variables
factorCols <- c("edu", "smoke", "drink", "chf", "cad",
                "hypertension", "hyperlipidemia", "dm",
                "ckd", "stroke", "status")
# Call function to fill missing values, where data is the data to be imputed
data <- fix_na(factorCols, data, 10)

write.csv(data, "imputed_data.csv")
```

```
### Install required packages
# install.packages("survminer") # Install survminer package
# install.packages("survival") # Install survival package
### Load required packages

res.cut <- surv_cutpoint(dd, # Data frame
                         time = "time_mon", # Time variable
                         event = "new_hf", # Status variable
                         variables = c("gcs", "gls", "grs") # Variables for optimal cutoff value,
single or multiple, provided as a vector
)
summary(res.cut)

#------Plotting optimal cutoff values
plot(res.cut,
     "gcs", # Plotting variable, single variable only
     palette = "npg" # Choose a palette, options include: "hue", "grey", "RdBu", "Blues", "npg",
"aaas", "lancet", "jco", "ucscgb", "uchicago", "simpsons", "rickandmorty" and more
)

plot(res.cut,
     "gls", # Plotting variable, single variable only
     palette = "npg" # Choose a palette, options include: "hue", "grey", "RdBu", "Blues", "npg",
"aaas", "lancet", "jco", "ucscgb", "uchicago", "simpsons", "rickandmorty" and more
)

plot(res.cut,
     "grs", # Plotting variable, single variable only
     palette = "npg" # Choose a palette, options include: "hue", "grey", "RdBu", "Blues", "npg",
"aaas", "lancet", "jco", "ucscgb", "uchicago", "simpsons", "rickandmorty" and more
)

#------------KM Plotting--------------------
#res.cat <- surv_categorize(res.cut) # Split data using optimal cutoff values, creating new data frame
#summary(res.cat) # Summarize the new data frame
#head(res.cat, 10) # View the first 10 rows of the new data frame
# Define cut line

dichotomize <- function (x, cutoff) {

  x_new <- ifelse(x > cutoff, 1, 0)
  x_new
}

## Plot survival curves
dd$GCS_2 <- dichotomize(dd$gcs, cutoff = -17.1177)
dd$GRS_2 <- dichotomize(dd$grs, cutoff = 34.0387)
dd$GLS_2 <- dichotomize(dd$gls, cutoff = -15.2089)
```

```
fit <- survfit(Surv(time_mon, new_hf) ~ GCS_2, data = dd) # Fit survival function with a single predictor and survival and status variables
fit1 <- coxph(Surv(time_mon, new_hf) ~ GCS_2, data = dd)
summary(fit1)
## Plot survival curves ---------GCS
ggsurvplot(fit,
        data = dd, # Data frame
        risk.table = TRUE, # Whether to show risk table
        conf.int = TRUE, # Whether to show confidence interval
        palette = "hue", # Choose a palette, options include: "hue", "grey", "RdBu", "Blues", "npg", "aaas", "lancet", "jco", "ucscgb", "uchicago", "simpsons", "rickandmorty" and more
        pval = TRUE, # Whether to show p-value
        pval.coord = c(50, 0.75), # p-value coordinates
        risk.table.height = 0.35, # Risk table height ratio
        risk.table.y.text = TRUE, # Whether to show risk table y-axis group names
        risk.table.y.text.col = TRUE,   # Whether risk table y-axis group names are colored
        censor.shape = "+",   # Censor symbol
        surv.median.line = "hv",   # Include median survival line, options: "hv": vertical + horizontal; "h": horizontal only; "v": vertical only; "none": no median line
        xlim = c(0, 200), # x-axis range
        break.x.by = 25, # x-axis interval
        ylim = c(0, 1), # y-axis range
        break.y.by = 0.25, # y-axis interval
        xlab = "Survival time (months)", # x-axis label
        ylab = "Survival probability", # y-axis label
        #title = "Image title", # Image title
        font.x = c(14, "black"), # x-axis label font size and color
        font.y = c(14, "black"), # y-axis label font size and color
        font.tickslab = c(12, "plain", "black"), # Coordinate scale font size and color
        legend = c(0.85, 0.95), # Legend position
        legend.title = "GCS", # Legend title
        legend.labs = c("high", "low"), # Legend group labels
        font.legend = c(12, "black"), # Legend font size and color
        font.title = c(15, "black") # Title size and color
)

#### Calculate model (grs) time AUC curve
f1 <- coxph(Surv(time_year, new_hf) ~ grs, data = d, x = TRUE, y = TRUE)

x1 = Score(list("grs" = f1),
        formula = Surv(time_year, new_hf) ~ 1,
        data = d, times = c(5:10),
        parallel = "as.registered",
        split.method = "bootcv", B = 100)
aucgraph <- plotAUC(x1)
summary(x1$plots)

#### Calculate model (gcs) time AUC curve
f2 <- coxph(Surv(time_year, new_hf) ~ gcs, data = d, x = TRUE, y = TRUE)
```

```
x2 = Score(list("gcs" = f2),
                    formula = Surv(time_year, new_hf) ~ 1,
                    data = d, times = c(5:10),
                    parallel = "as.registered",
                    split.method = "bootcv", B = 100)
aucgraph <- plotAUC(x2)
summary(x2)

# Plotting forest plots
forestplot(labeltext = as.matrix(data[, 1:3]),
                    mean = data$HR, # HR values
                    lower = data$LowerCI, # Confidence interval
                    upper = data$UpperCI, # Confidence interval
                    is.summary = c(TRUE, TRUE, FALSE, FALSE, TRUE, FALSE, FALSE, TRUE,
FALSE, FALSE, TRUE, FALSE, FALSE, TRUE, FALSE, FALSE), # Corresponding factor part
                    xticks = c(0.2, 0.6, 1, 1.4, 1.8),
                    lwd.xaxis = 2,
                    clip = c(0.6, 1.2),
                    zero = 1, # Vertical line position
                    lwd.zero = 2, # Vertical line width
                    lineheight = 'auto', # Line height, auto
                    colgap = unit(8, 'mm'), # Column gap, affects figure width
                    boxsize = 0.2, # Black square size
                    lwd.ci = 2, # Confidence interval horizontal line width
                    graph.pos = 4,
                    col = fpColors(all.elements = 'black')) # Set font color

#install.packages("ggstatsplot")

#------------------------GCS-------------------------
#----------------GCS -LVEF----------
ggscatterstats(
   data    = dd,
   x        = GCS,
   y        = LVEF,
   xlab    = "GCS",
   ylab    = "LVEF",)
```

Stata Code

```
stset time , failure ( status )
continuous
stepwise, pr(.05) :stcox age sex eth edu bmi dbp sbp hba1c ldl drinking smoking cad hypertension eGFR DM_time drug grs
stepwise, pr(.05) :stcox age sex eth edu bmi dbp sbp hba1c ldl drinking smoking cad hypertension eGFR DM_time drug gcs
stepwise, pr(.05) :stcox age sex eth edu bmi dbp sbp hba1c ldl drinking smoking cad hypertension eGFR DM_time drug gls
categorical
stepwise, pr(.05) :stcox age sex eth edu bmi dbp sbp hba1c ldl drinking smoking cad hypertension eGFR DM_time drug rs
stepwise, pr(.05) :stcox age sex eth edu bmi dbp sbp hba1c ldl drinking smoking cad hypertension eGFR DM_time drug cs
stepwise, pr(.05) :stcox age sex eth edu bmi dbp sbp hba1c ldl drinking smoking cad hypertension eGFR DM_time drug ls
```
